# Association between sevelamer use and outcomes in acute kidney injury with hyperphosphataemia: evidence from the MIMIC-IV

**DOI:** 10.3389/fphar.2026.1776446

**Published:** 2026-04-29

**Authors:** Shuzhen Suo, Qilin Yang, Weiyan Chen, Liya Luo, Yongkang Song, Chunxiang Ling, Jianzhong Wang, Deliang Wen, Changbo Liu

**Affiliations:** 1 Department of Critical Care, The Fourth Affiliated Hospital of Guangzhou Medical University, Guangzhou, China; 2 Department of Critical Care, The Second Affiliated Hospital of Guangzhou Medical University, Guangzhou, China

**Keywords:** acute kidney injury, hyperphosphataemia, mimic iv, mortality, propensity-score, sevelamer

## Abstract

**Background:**

Hyperphosphataemia is consistently linked to adverse clinical outcomes in Acute Kidney Injury (AKI). Sevelamer, a non-calcium phosphate binder widely used in chronic kidney disease (CKD), has not been adequately evaluated for efficacy in AKI.

**Methods:**

We performed a retrospective cohort analysis in the MIMIC- IV database. The association between sevelamer exposure and 28- day all-cause mortality was estimated using multivariable Cox models and propensity score matching (PSM).

**Results:**

After adjustment for baseline excoriates, sevelamer use was associated with a 48% relative lower in 28- day mortality (adjusted HR 0.52; 95% CI 0.39–0.68; P < 0.001). Findings were consistent across propensity-score methods. Overlap-weighted Kaplan–Meier curves showed higher cumulative survival in the sevelamer group (P < 0.001). In two-part hurdle model analyses, sevelamer use was associated with significantly higher odds of having any ventilator-free days (OR = 2.53, *P* < 0.001), any ICU-free days (OR = 1.64, *P* = 0.004), and any vasopressor-free days (OR = 2.74, *P* < 0.001) within 28 days. Subgroup analyses suggested greater benefit among non-diabetic patients (HR 0.42 vs. 0.79; interaction P = 0.023) and in patients who commenced renal replacement therapy (RRT) within 1 day of admission (HR 0.27 vs. 0.63; interaction P = 0.007).

**Conclusion:**

In this ICU cohort of AKI patients with Hyperphosphataemia, sevelamer use was associated with substantially lower 28- day mortality. Moreover, sevelamer administration was associated with a clinically significant increase in the likelihood of liberation from mechanical ventilation, ICU discharge, and discontinuation of vasopressor support. These findings broaden the clinical indications of sevelamer and propose a novel therapeutic strategy for the management of AKI-associated hyperphosphatemia.

## 1 Introduction

AKI (Acute kidney injury) is a common and severe complication in intensive care units (ICU), with reported incidence between 20%–50% and in-hospital mortality frequently in the range of 40%–60% (Hoste et al., 2015; Kaddourah et al., 2017; Cerda et al., 2025). Hyperphosphataemia, a principal manifestation of disturbed mineral metabolism, is an established adverse prognostic indicator in AKI (Hamano and Fukagawa, 2016). Hyperphosphataemia affects approximately 30%–70% of patients with AKI and is associated with increased mortality (Leaf et al., 2013; Jung et al., 2016).

Sevelamer is a non-calcium, non-metal polymeric phosphate binder that sequesters anionic phosphate in the gut to form insoluble complexes excreted in faeces, thereby lowering intestinal phosphate uptake and serum phosphate (Slatopolsky et al., 1999; Malberti, 2013). In clinical trials involving CKD populations, sevelamer has demonstrated efficacy and an acceptable safety profile, notably reducing the rate of vascular calcification and slowing the deterioration of renal function (Patel et al., 2016; Wang et al., 2022). However, randomised and robust observational data on sevelamer specifically in AKI are scarce.

To address this evidence gap, we analysed MIMIC- IV v3.1, a large, high-quality ICU electronic health record repository (2008–2019), to evaluate the association between sevelamer exposure and outcomes in AKI patients with hyperphosphataemia.

## 2 Methods

### 2.1 Design and data source

We conducted a retrospective cohort study using de-identified data from MIMIC- IV v3.1. It is a large single-centre critical care electronic health record repository curated by the MIT Laboratory for Computational Physiology in collaboration with Beth Israel Deaconess Medical Center. The source data were collected during routine clinical care (2008–2019) and subsequently assembled to support reproducible clinical, epidemiologic and methodological research in critical care. The study conformed to the Declaration of Helsinki; institutional review board approval with waiver of consent was obtained (MIT protocol 0403000206). All investigators completed human-subjects protection training. One author QilingYang obtained approval to exploit the database (certification number 7634793). This study adheres to the guidelines of the Strengthening the Reporting of Observational Studies in Epidemiology (STROBE) statement.

### 2.2 Cohort selection

**FIGURE 1:**
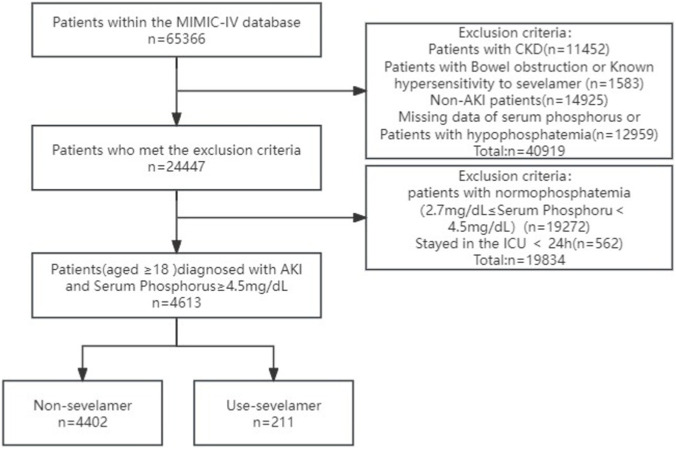
The flow chart of the study.

Adult ICU admissions were screened consecutively. Inclusion and exclusion criteria were applied in the following order: (1) identification of AKI per KDIGO criteria during the index ICU stay; (2) availability of at least one serum phosphate measurement during the index ICU admission; (3) exclusion of patients with pre-existing chronic kidney disease (CKD, see below); (4) exclusion of admissions with bowel obstruction or documented sevelamer allergy; (5) exclusion of admissions with missing serum phosphate or documented hypophosphataemia; and (6) exclusion of ICU stays <24 h. The final analytic cohort comprised 4,613 adults (≥18 years) with AKI and serum phosphate ≥4.5 mg/dL at ICU admission. Patients were classified by sevelamer exposure during the ICU stay: sevelamer (n = 211) versus no sevelamer (n = 4,402) (Figure 1).

### 2.3 Definitions and exposure

AKI was defined using KDIGO criteria applied to serum creatinine and urine-output in MIMIC-IV (creatinine rise ≥0.3 mg/dL within 48 h or ≥1.5× baseline within 7 days; urine output <0.5 mL/kg/h for 6 h).”phosphate at ICU admission” was operationalized as the first serum phosphate measurement recorded during the index ICU admission. Hyperphosphataemia was defined as serum phosphate ≥4.5 mg/dL.Chronic kidney disease (CKD) was defined operationally as either (a) documentation of CKD in the problem list or prior encounter diagnoses (ICD codes for CKD) before the index ICU admission, or (b) evidence of maintenance dialysis prior to the index admission (procedure/order records indicating chronic outpatient dialysis). Where both sources were available, either criterion classified the admission as pre-existing CKD. Sevelamer exposure was identified from prescription records; duration was measured from first to last administration. We defined ICU admission as time zero for all adult (≥18) patients with AKI and hyperphosphataemia (serum phosphate ≥4.5 mg/dL) and classified sevelamer exposure as initiation within the first 72 h.

### 2.4 Covariates

Baseline covariates included demographics, mean vital signs in the first 24 h, worst laboratory values in the first 24 h (e.g., BUN, creatinine, electrolytes), severity scores (CCI, APSIII, SAPSII, OASIS, SOFA), comorbidities (ICD codes), and initiation of RRT within day 1. Demographics captured patient age and sex. Laboratory parameters comprised hemoglobin, platelet count, white blood cell count, anion gap, serum bicarbonate, blood urea nitrogen (BUN), serum creatinine, serum calcium, chloride, sodium, potassium, magnesium, phosphate and blood glucose. Illness severity was quantified using several scoring systems: Charlson Comorbidity Index (CCI), Acute Physiolog Score III (APSIII), Simplified Acute Physiology Score II (SAPSII), Oxford Acute Severity of Illness Score (OASIS) and Sequential Organ Failure Assessment (SOFA). Comorbid conditions—identified via ICD- 9 and ICD- 10 diagnostic codes—included diabetes, myocardial infarction, congestive heart failure, peripheral vascular disease, cerebrovascular disease, chronic pulmonary disease and dementia. Treatment variables noted whether renal replacement therapy (RRT) was commenced within the first day of the ICU stay.

### 2.5 Outcomes

The primary outcome was 28- day all-cause mortality. Secondary outcomes were ventilator-free days, ICU- free days and vasopressor-free days within 28 days; patients who died within 28 days were assigned 0 days for these endpoints. Ventilator-free days were defined as 28 minus the total days spent on mechanical ventilation; ICU- free days as 28 minus the ICU length of stay; and vasopressor-free days as 28 minus the cumulative days of vasopressor therapy (norepinephrine, epinephrine, dopamine, vasopressin, or phenylephrine). Patients who died within 28 days were assigned a value of zero for all secondary endpoints. Follow-up continued until death or day 28, and database records confirmed there were no losses to follow-up.

### 2.6 Statistical analysis

Continuous variables were summarised as mean ± SD or median (IQR); categorical variables as counts and percentages. Baseline balance was evaluated by standardised mean differences (SMD). The Boruta random-forest wrapper was used for feature selection among baseline variables. Primary analyses employed multivariable Cox proportional hazards models adjusting for all baseline covariates; proportional hazards assumptions were assessed using Schoenfeld residuals. To reduce confounding, propensity scores were estimated with logistic regression including all covariates, and four approaches were applied: PS- adjusted Cox, 1:4 nearest-neighbour matching with caliper (0.2 × SD of logit PS), partial adjustment weighting and overlap weighting. Post-matching balance was checked by SMD. Subgroup analyses tested interactions between exposure and prespecified factors (age, AKI stage, diabetes, cardiovascular comorbidities, early RRT). The subgroup variables listed were prespecified and confirm no additional subgroup tests were performed *post hoc*. Secondary endpoints were analysed with two-part hurdle model. Survival functions were estimated by Kaplan–Meier methods and compared with log-rank tests. All the analyses were performed with the statistical software packages R 3.3.2 (http://www.R-project.org, The R Foundation) and Free Statistics software versions 2.1. Two-sided P < 0.05 denoted statistical significance.

## 3 Results

### 3.1 Baseline characteristics of the study population

Demographic and clinical characteristics of the final cohort are summarized in Table 1. Prior to matching, patients who received sevelamer differed markedly from controls: they were younger (59.64years vs. 63.52 years; SMD = 0.245), more often male (68.2% vs. 54.7%; SMD = 0.280) had worse renal indices (peak creatinine 4.58 vs. 2.01 mg/dL; SMD = 0.937; peak BUN 68.21 vs. 36.88 mg/dL; SMD = 0.913), higher physiologic severity (APSIII 85.89 vs. 63.68; SMD = 0.761) and more frequent early RRT (34.6% vs. 8.5%; SMD = 0.669). After 1:4 matching (treated n = 186, controls n = 744) baseline covariates achieved close balance: age (60.30 vs. 60.52; SMD = 0.015), sex (male 65.6% vs. 66.7%; SMD = 0.023) and RRT within day- 1 (26.9% vs. 27.4%; SMD = 0.012), and all reported variables met the SMD <0.1 criterion, supporting comparability for outcome analyses. (Table 1) Propensity-score ROC curves and Love plots showing standardized mean differences before and after adjustment are provided in the Supplement (Supplementary Figures 1, 2).

**TABLE 1:** Baseline characteristics of patients before and after propensity score matching.

Characteristics	Before matching			After matching		
	No sevelamer	Sevelamer	SMD	No sevelamer	Sevelamer	SMD
	(n = 4,402)	(n = 211)		(n = 744)	(n = 186)	
Demographics						
Age, years	63.52 ± 16.32	59.64 ± 15.32	0.245	60.52 ± 15.21	60.30 ± 15.30	0.015
Male, n (%)	2,410 (54.7)	144 (68.2)	0.280	496 (66.7)	122 (65.6)	0.023
Vital signs						
Heart rate, bpm	88.39 ± 17.00	90.18 ± 17.40	0.104	90.31 ± 17.14	90.00 ± 17.40	0.018
Mean blood pressure, mmHg	77.41 ± 10.67	77.25 ± 11.75	0.015	76.37 ± 11.53	77.00 ± 11.24	0.056
Respiratory rate,/min	20.04 ± 4.42	21.80 ± 5.02	0.372	22.01 ± 5.07	21.65 ± 4.99	0.072
Temperature, °C	36.78 ± 0.62	36.86 ± 0.73	0.111	36.86 ± 0.65	36.83 ± 0.74	0.037
SpO2, %	96.63 ± 2.61	96.48 ± 2.18	0.062	96.48 ± 2.52	96.48 ± 2.25	0.002
Laboratory parameters						
Hemoglobin (min), g/dL	9.81 ± 2.46	9.13 ± 2.39	0.279	9.13 ± 2.33	9.25 ± 2.45	0.049
Platelet count (min), ×103/μL	181.44 ± 117.27	174.02 ± 125.77	0.061	163.22 ± 129.34	166.84 ± 118.60	0.029
WBC count (max), ×103/μL	17.34 ± 13.33	22.05 ± 38.34	0.164	20.96 ± 23.40	20.55 ± 30.77	0.015
Anion gap (max), mEq/L	18.61 ± 6.46	21.55 ± 6.03	0.472	20.93 ± 6.89	21.27 ± 6.08	0.052
Bicarbonate (min), mEq/L	19.74 ± 5.82	17.94 ± 5.08	0.330	18.46 ± 6.35	18.18 ± 5.15	0.049
BUN (max), mg/dL	36.88 ± 27.38	68.21 ± 40.04	0.913	59.76 ± 36.60	63.17 ± 37.15	0.092
Calcium (min), mg/dL	8.01 ± 0.93	7.83 ± 0.98	0.181	7.86 ± 0.98	7.81 ± 0.94	0.044
Chloride (min), mEq/L	100.47 ± 6.72	97.30 ± 6.98	0.463	97.38 ± 8.53	97.48 ± 6.95	0.013
Creatinine (max), mg/dL	2.01 ± 1.97	4.58 ± 3.34	0.937	3.53 ± 2.79	4.14 ± 2.94	0.214
Sodium (min), mEq/L	136.08 ± 5.53	134.32 ± 6.12	0.301	134.08 ± 7.03	134.46 ± 6.12	0.057
Potassium (max), mEq/L	4.95 ± 0.93	5.16 ± 1.02	0.218	5.13 ± 1.01	5.13 ± 1.02	0.002
Magnesium, mg/dL	2.11 ± 0.60	2.25 ± 0.46	0.258	2.23 ± 0.56	2.23 ± 0.47	0.001
Phosphate, mg/dL	5.59 ± 1.52	6.73 ± 1.89	0.669	6.35 ± 2.23	6.49 ± 1.68	0.075
Glucose, mg/dL	152.59 ± 53.96	150.73 ± 49.77	0.036	151.79 ± 56.74	152.86 ± 50.67	0.020
Severity scores						
Charlson comorbidity index	5.46 ± 2.75	5.60 ± 2.97	0.048	5.71 ± 2.70	5.66 ± 3.01	0.017
APSIII	63.68 ± 30.22	85.89 ± 28.12	0.761	87.94 ± 30.25	84.61 ± 27.73	0.115
SAPSII	43.86 ± 16.25	51.04 ± 15.42	0.454	51.68 ± 16.82	50.90 ± 15.25	0.049
OASIS	36.62 ± 10.04	40.68 ± 10.20	0.401	41.45 ± 10.37	40.51 ± 10.15	0.092
SOFA	6.75 ± 4.10	9.24 ± 3.72	0.636	9.59 ± 4.30	9.25 ± 3.81	0.083
Comorbidities, n (%)						
Diabetes mellitus	1,255 (28.5)	62 (29.4)	0.019	219 (29.4)	54 (29.0)	0.009
Myocardial infarction	748 (17.0)	39 (18.5)	0.039	131 (17.6)	35 (18.8)	0.031
Congestive heart failure	1,256 (28.5)	66 (31.3)	0.060	238 (32.0)	57 (30.6)	0.029
Peripheral vascular disease	514 (11.7)	29 (13.7)	0.062	107 (14.4)	26 (14.0)	0.012
Cerebrovascular disease	513 (11.7)	27 (12.8)	0.035	92 (12.4)	24 (12.9)	0.016
Chronic pulmonary disease	1, 194 (27.1)	56 (26.5)	0.013	222 (29.8)	51 (27.4)	0.054
Dementia	116 (2.6)	3 (1.4)	0.086	13 (1.7)	3 (1.6)	0.010
Treatment						
RRT within 1 day, n (%)	375 (8.5)	73 (34.6)	0.669	204 (27.4)	50 (26.9)	0.012

Data are presented as mean ± standard deviation for continuous variables and n (%) for categorical variables.

Abbreviations: SMD, standardized mean difference; SpO_2_, oxygen saturation; WBC, white blood cell; BUN, blood urea nitrogen; APSIII, Acute Physiology Score III; SAPSII, Simplified Acute Physiology Score II; OASIS, oxford acute severity of illness score; SOFA, sequential organ failure assessment; RRT, renal replacement therapy.

SMD <0.1 indicates good balance between groups.

### 3.2 Feature selection

Boruta was applied as a preliminary feature screening tool to identify clinically and statistically relevant predictors of 28-day mortality from the full set of baseline variables. The final multivariable adjustment set was determined by combining Boruta-selected important variables with clinically relevant confounders predefined based on existing literature and clinical expertise, rather than relying solely on algorithmic output. Furthermore, we have added a formal collinearity assessment section:Variance inflation factors (VIF) were calculated for all variables in the final adjustment model (Supplementary Table 1).

**FIGURE 2:**
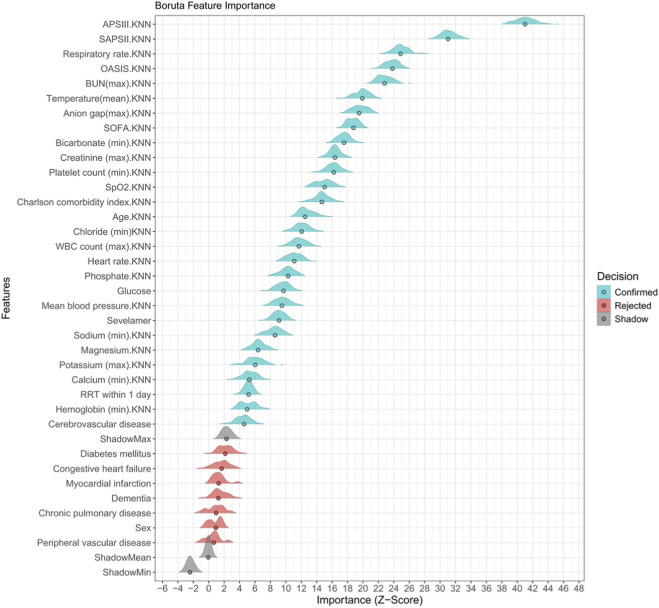
Borda feature importance ranking of clinical features in AKI patients with hyperphosphataemia.

A strict threshold of VIF ≥10 was applied to exclude severely collinear variables. No significant multicollinearity was detected among the final adjustment variables, ensuring the stability and reliability of regression estimates. Boruta identified 26 of 37 baseline variables as important predictors of 28- day mortality; composite severity scores ranked highest, followed by respiratory rate, BUN, temperature, anion gap, bicarbonate and creatinine. Sevelamer exposure was flagged as an influential variable, suggesting independent prognostic relevance (Figure 2).

### 3.3 Primary outcome

Overall 28- day mortality was 26.5% (1,224/4,613). Unadjusted mortality did not differ materially between groups (27.0% vs. 26.5%). Crude Cox analysis showed no significant association (HR 0.97.95%CI:0.74–1.26,P = 0.817). After multivariable adjustment for 37 covariates, sevelamer was associated with lower 28- day mortality (HR 0.52.95% CI: 0.39–0.68,P < 0.001), corresponding to a 48% relative risk reduction. Sensitivity analyses using PS- adjusted Cox (HR 0.66.95% CI: 0.50–0.87,P = 0.003), 1:4 matching (HR 0.58.95% CI: 0.43–0.77,P < 0.001), partial adjustment weighting (HR0.55.95% CI: 0.40–0.77,P < 0.001) and overlap weighting (HR 0.59.95% CI: 0.41–0.85,P < 0.001) yielded consistent protective estimates (HR range 0.55–0.66) (Table 2).

**TABLE 2:** Association between sevelamer use and 28-day mortality in critically ill patients.

Exposure/Analysis method	Mortality, no. (%) events/At risk	Hazard ratio (95% CI)	P value
No sevelamer use	1, 167/4,402 (26.5)	—	—
Sevelamer use	57/211 (27.0)	—	—
Crude analysis	—	0.97 (0.74–1.26)	0.817
Multivariable-adjusted model^a^	—	0.52 (0.39–0.68)	<0.001
Propensity score–based analyses			
• Adjusted for propensity score^b^	—	0.66 (0.50–0.87)	0.003
• Propensity score matching^c^	—	0.58 (0.43–0.77)	<0.001
• Partial adjustment weighting^d^	—	0.55 (0.40–0.77)	<0.001
• Overlap weighting^e^	—	0.59 (0.41–0.85)	<0.001

^a^ Adjusted for age, sex, vital signs (heart rate, mean arterial pressure, respiratory rate, temperature,SpO2), laboratory values (haemoglobin, platelets, WBC, anion gap, bicarbonate; BUN, creatinine, calcium, chloride, sodium, potassium, magnesium, phosphate, glucose), severity scores (Charlson Comorbidity Index, APSIII, SAPSII, OASIS, SOFA), and comorbidities (diabetes, myocardial infarction, heart failure, peripheral vascular disease, cerebrovascular disease, chronic pulmonary disease, dementia).

^b^ Multivariable Cox model with additional adjustment for propensity score as a continuous covariate.

^c^ Cox model stratified on matched pairs; 930 patients included (186 sevelamer users, 744 non-users).

^d^ Weighted Cox model using partial adjustment (PA) propensity score weights.

^e^ Weighted Cox model using overlap weighting (OW) to estimate average treatment effect in the overlap population.

### 3.4 Survival and secondary outcomes

**FIGURE 3:**
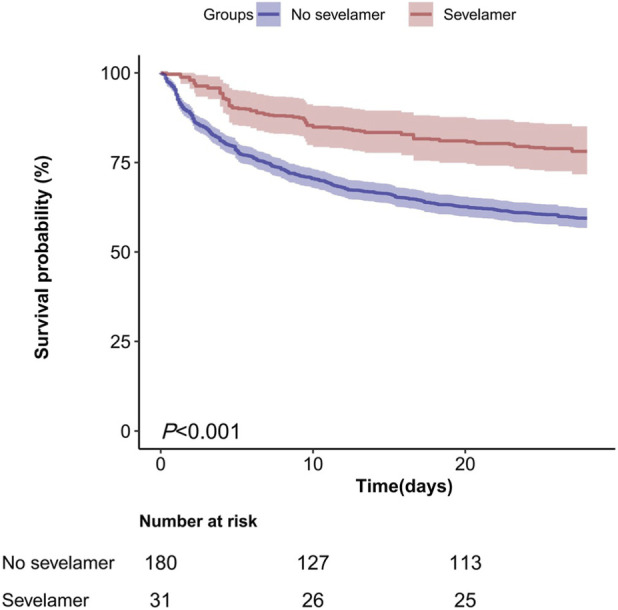
The Kaplan–Meier Curves for 28-Day Mortality in AKI Patients with Hyperphosphataemia: Sevelamer vs. Non-Sevelamer Groups.

The Overlap-weighted Kaplan–Meier survival analysis revealed significant differences in survival probabilities among the two groups (log-rank P < 0.001). The curves demonstrated early and sustained separation in favour of sevelamer, with the largest divergence during the first week and an approximate absolute survival advantage of 25 percentage points at day 28 (Figure 3).

**FIGURE 4:**
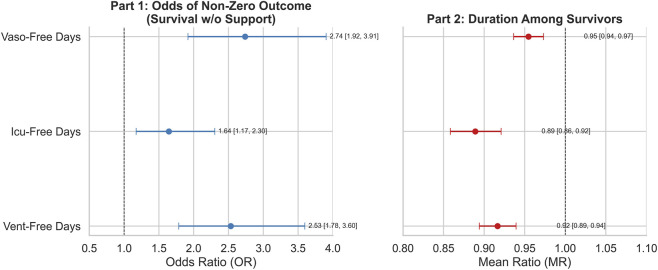
The two-part Hurdle model of secondary outcomes: ventilator-free days, ICU-free days and vasopressor-free days.

Given that the secondary outcomes exhibited right-skewed, zero-inflated distributions (Supplementary Figures 3–5), we therefore employed a two-part hurdle model to analyze these outcomes (Figure 4). It revealed distinct but clinically meaningful effects:Survival/weaning benefit (Part 1): Sevelamer significantly increased the odds of achieving non-zero ventilator-free (OR = 2.53, p < 0.001), ICU-free (OR = 1.64, p = 0.004), and vasopressor-free days (OR = 2.74, p < 0.001); Recovery duration (Part 2): Among survivors, sevelamer was associated with slightly shorter mean free days (mean ratio = 0.89–0.95, all p < 0.001), reflecting survivor bias rather than a detrimental effect.

### 3.5 Sensitivity analysis and subgroup analyses

We performed sensitivity analyses focused on early sevelamer exposure. we examined its association with outcomes using an multivariable Cox analysis. After adjustment for the prespecified covariates, sevelamer use was associated with a lower hazard of death (adjusted HR 0.69; 95% CI 0.49–0.98; P = 0.04), corresponding to a 31% relative reduction in the instantaneous risk of 28-day mortality. These sensitivity analyses produced results that are consistent with the study’s primary findings (Supplementary Table 2).

Subgroup analyses showed heterogeneity of effect. Sevelamer use was associated with reduced 28- day mortality across most subgroups. The effect reached statistical significance in patients aged ≥65 years (adjusted HR 0.44, 95% CI 0.29–0.67, P < 0.001) and in those with AKI stage 3 (HR 0.44, 95% CI 0.31–0.62, P < 0.001). A larger benefit was observed in non-diabetic patients (HR 0.42, 95% CI 0.30–0.59, P < 0.001) than in patients with diabetes (HR 0.79, 95% CI 0.48–1.30, P = 0.35), with evidence of effect modification (P for interaction = 0.023). The mortality reduction was particularly marked in patients receiving RRT within 1 day (HR 0.27, 95% CI 0.15–0.51, P < 0.001; P for interaction = 0.007). Other interaction tests were non-significant, indicating generally consisten associations across the remaining strata. (Table 3) The forest plot for subgroup analyses is presented Supplementary Figure 6.

**TABLE 3:** Subgroup analyses of the association between sevelamer use and 28-day mortality.

Subgroup	Total no.	Deaths, no. (%)	Adjusted HR (95% CI)^a^	P-value	P For interaction^b^
Age group	​	​	​	​	0.099
≥ 65 years	128	25 (19.5)	0.44 (0.29–0.67)	<0.001	​
< 65 years	83	32-(38.6)	0.69 (0.47–1.01)	0.053	​
AKI stage (7-day)	​	​	​	​	0.275
Stage 1	36	7-(19.4)	0.53 (0.22–1.30)	0.168	​
Stage 2	34	13-(38.2)	0.65 (0.35–1.19)	0.163	​
Stage 3	141	37-(26.2)	0.44 (0.31–0.62)	<0.001	​
Diabetes mellitus	​	​	​	​	0.023
No	149	38-(25.5)	0.42 (0.30–0.59)	<0.001	​
Yes	62	19-(30.6)	0.79 (0.48–1.30)	0.348	​
Myocardial infarction	​	​	​	​	0.539
No	172	46-(26.7)	0.53-(0.39–0.72)	<0.001	​
Yes	39	11-(28.2)	0.34 (0.18–0.65)	0.001	​
Congestive heart failure	​	​	​	​	0.210
No	145	42-(29.0)	0.60 (0.44–0.84)	0.003	​
Yes	66	15-(22.7)	0.43 (0.25–0.73)	0.002	​
Peripheral vascular disease	​	​	​	​	0.796
No	182	50-(27.5)	0.52-(0.38–0.69)	<0.001	​
Yes	29	7-(24.1)	0.40 (0.17–0.95)	0.038	​
Cerebrovascular disease	​	​	​	​	0.357
No	184	46-(25.0)	0.48-(0.35–0.65)	<0.001	​
Yes	27	11-(40.7)	0.60 (0.29–1.22)	0.158	​
Chronic pulmonary disease	​	​	​	​	0.254
No	155	40-(25.8)	0.46-(0.33–0.64)	<0.001	​
Yes	56	17-(30.4)	0.57 (0.34–0.98)	0.043	​
RRT within 1 day	​	​	​	​	0.007
No	138	45-(32.6)	0.63 (0.46–0.86)	0.004	​
Yes	73	12-(16.4)	0.27 (0.15 to −0.51)	<0.001	​

^a^ Adjusted hazard ratios derived from Cox proportional hazards models, stratified by subgroup, with adjustment for age, sex, vital signs (heart rate, mean arterial pressure, respiratory rate, temperature, SpO2), laboratory parameters (haemoglobin, platelet count, white blood cell count, anion gap, bicarbonate, blood urea nitrogen, creatinine, calcium, chloride, sodium, potassium, magnesium, phosphate, glucose), severity scores (Charlson Comorbidity Index, APSIII, SAPSII, OASIS, SOFA), and comorbidities (diabetes mellitus, myocardial infarction, congestive heart failure, peripheral vascular disease, cerebrovascular disease, chronic pulmonary disease, dementia).

^b^ P value for interaction tests whether the effect of sevelamer on 28-day mortality differs significantly across levels of the subgroup variable in the adjusted model.

Reference group: Patients not receiving sevelamer within each subgroup.

## 4 Discussion

Using the large, real-world MIMIC- IV 3.1 database, this study is, to our knowledge, the first to systematically assess sevelamer’s clinical utility in a cohort of patients with AKI complicated by hyperphosphatemia Multivariable adjustment showed sevelamer treatment was associated with a 48% reduction in 28- day mortality. This association was robust across four propensity-score methods, arguing against a major influence of analytic choice or residual confounding. The two-part hurdle model separates two clinically distinct issues: the probability of survival and liberation from organ support, and the recovery duration among survivors. Applying this model, sevelamer was associated with higher odds of survival/weaning (OR > 1) but did not consistently shorten recovery time in those who survived. Apparent changes in recovery duration likely reflect survivor-selection bias—sevelamer salvages patients who would otherwise have died (and thus been recorded as 0 days), altering the survivor pool—rather than a true acceleration of convalescence. Hence, the hurdle analysis reveals a clinically meaningful benefit (a reduction in the worst-case outcome of death or prolonged organ-support dependence). Predefined subgroup analyses identified effect modification: non-diabetic patients and those receiving early RRT derived greater benefit. The subgroup findings are exploratory and that the interaction signals (diabetes and early RRT) do not survive strict correction for multiple comparisons. We will emphasise that the RRT interaction had the smallest nominal p-value but that even this result requires validation in independent cohorts or ideally in randomized studies.

Our findings corroborate prior epidemiological work linking hyperphosphatemia to adverse outcomes, importantly, we provide intervention-based clinical evidence. Jung et al., analysing a Korean national registry, reported that raised serum phosphate independently predicted in-hospital mortality in patients with AKI, although that study was observational and did not assess therapeutic interventions (Jung et al., 2016). Likewise, the prospective cohort by Leaf et al. linked elevated phosphate and increased FGF23 to worse AKI prognosis, but did not evaluate phosphate-lowering strategies (Leaf et al., 2013). By comparing survival between patients treated with sevelamer and untreated patients, our study delivers the first interventional signal that active reduction of phosphate may improve outcomes in AKI.

The independent trial in CKD reported reduced cardiovascular mortality with sevelamer (HR 0.38) (Di Iorio et al., 2012), a magnitude broadly comparable to our observed reduction in all-cause mortality (HR 0.52). The clinical contexts, however, differ markedly: phosphate excess in CKD accrues over months to years, whereas in AKI it emerges rapidly and can become life-threatening within days (Quinn et al., 2020). Importantly, the protective effect in our cohort appeared within the first week, contrasting with the delayed benefits seen in CKD and implying distinct temporal dynamics and mechanisms in acute illness (Natale et al., 2026). Unlike Chertow et al., who concentrated on vascular calcification (Chertow et al., 2002), we used mortality as the primary endpoint, enhancing clinical relevance. The observed association between sevelamer and reduced vasopressor exposure—not previously reported in CKD cohorts—may reflect a specific haemodynamic advantage in the acute setting.

Chertow et al. primarily examined progression of vascular calcification in haemodialysis patients (Chertow et al., 1999; Nelson et al., 2020), whereas our study adopts mortality as the primary endpoint, offering more direct clinical applicability. We found sevelamer use to be associated with a shorter duration of vasopressor therapy—an observation not reported in CKD cohorts—which may indicate a specific haemodynamic benefit in the context of acute illness.

Sevelamer’s benefits in AKI likely come from both phosphate-dependent and phosphate-independent effects (Habbous et al., 2017; Basutkar et al., 2022). Phosphate-dependen: by binding intestinal phosphate and lowering serum levels, sevelamer may quickly reduce phosphate-driven vascular and cardiac injury, improve endothelial function, restore NO- mediated vasodilation, and lessen vasopressor needs by reversing “phosphotoxic” myocardial dysfunction (Chertow et al., 1999; Sprague, 2007; Vlassara et al., 2012a; Chennasamudram et al., 2013; Rastogi, 2013; Yubero-Serrano et al., 2015; Habbous et al., 2017; Basutkar et al., 2022). Phosphate-independent: sevelamer adsorbs intestinal endotoxin (LPS) and uraemic toxins such as AGEs, reducing endotoxaemia and systemic inflammation—especially relevant in sepsis-associated AKI (Brandenburg et al., 2010; Cozzolino et al., 2012; Vlassara et al., 2012b; Chennasamudram et al., 2013; Yang et al., 2018; Cernaro et al., 2023). Its lipid-lowering effects and the alkali load from carbonate formulations may further protect the heart and help correct metabolic acidosis. Taken together, these pleiotropic actions may reduce endothelial injury, myocardial depression, and inflammatory dysregulation in AKI, particularly when systemic inflammation is present.

We observed marked subgroup heterogeneity in sevelamer’s protective association in AKI with hyperphosphataemia. The survival benefit was greatest in non-diabetic patients. Diabetes-related vascular disease, chronic inflammation, impaired gut motility, and disturbed phosphate handling may blunt sevelamer’efficacy.This pattern suggests that diabetic patients with AKI and hyperphosphataemia may need a broader strategy—such as tighter glycaemic control and more aggressive infection management—rather than relying on phosphate lowering alone. The largest relative mortality reduction (73%) occurred in patients who started RRT within 24 h, suggesting that in the sickest patients, adjunctive sevelamer may help control interdialytic phosphate and prevent rebound hyperphosphataemia.

## 5 Limitations

Several limitations warrant cautious interpretation. First, despite employing multiple propensity-score techniques and multivariable adjustment, residual unmeasured confounding cannot be excluded. Second, the absence of precise timestamps for sevelamer administration and AKI onset precluded time-dependent modelling and prevented assessment of early versus late initiation. Furthermore, classifying exposure as any sevelamer receipt during the ICU admission without a time-varying specification may introduce immortal-time bias and confounding by indication; these biases are not corrected by the adjustment methods used and could have led to an overestimation of benefit. Future studies with high-resolution temporal data should apply time-varying Cox models and prespecified landmark analyses to corroborate our findings. Finally, because the cohort was drawn from a single tertiary academic centre, differences in case mix, practice patterns and resources may limit generalisability, and external validation is required.

## 6 Conclusion

In this ICU cohort of patients with AKI and hyperphosphataemia, sevelamer therapy was associated with markedly lower 28-day mortality and a clinically meaningful increased probability of liberation from mechanical ventilation, ICU discharge, and vasopressor support cessation.

## Data Availability

The raw data supporting the conclusions of this article will be made available by the authors, without undue reservation.
