# Supplementary figures and images for: Association between sevelamer use and outcomes in acute kidney injury with hyperphosphataemia: evidence from the MIMIC-IV
Source: Front Pharmacol. 2026 Apr 29;17:1776446. doi: 10.3389/fphar.2026.1776446 (PMC13167594; doi:10.3389/fphar.2026.1776446)

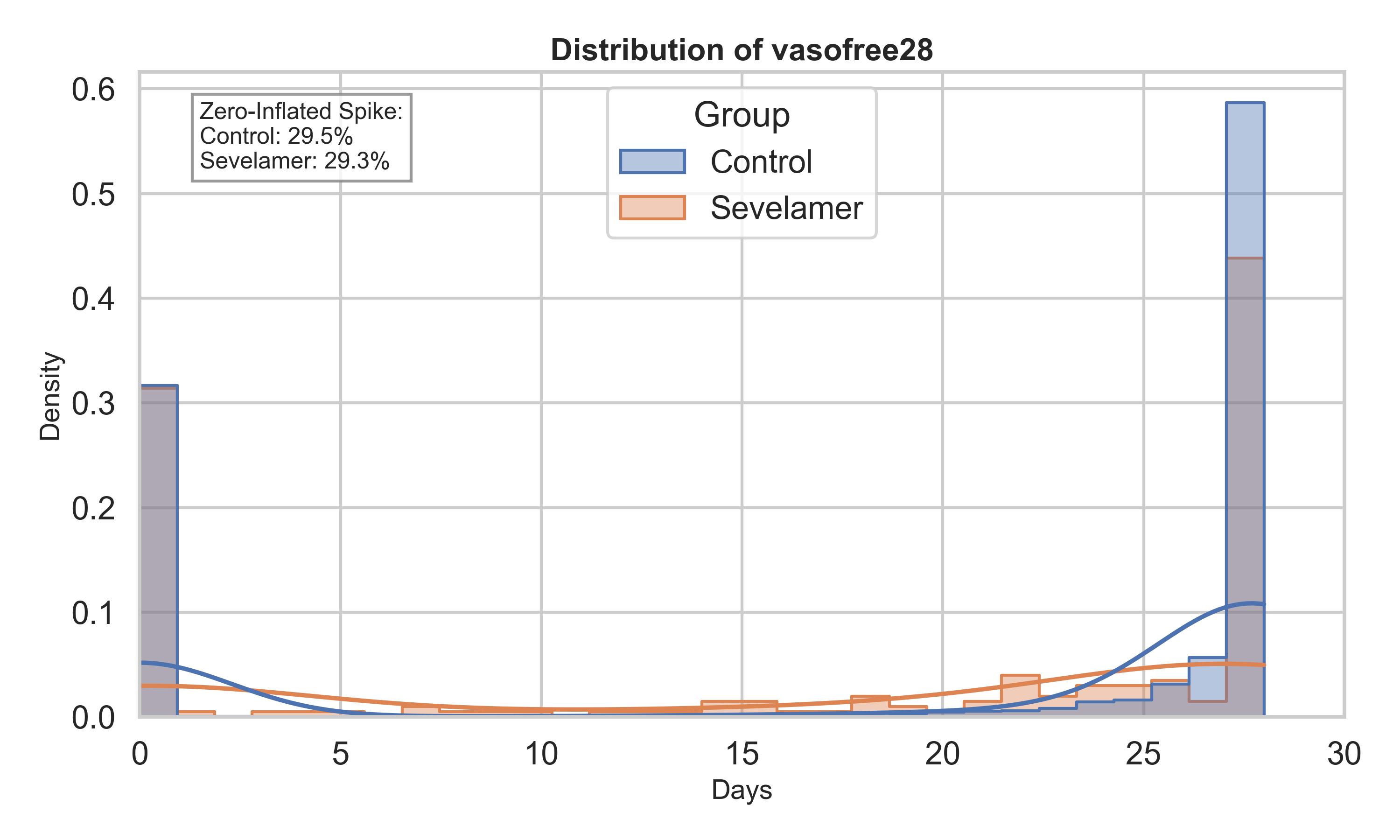

Supplement: Supplementary file 1 [file Image3.jpeg]

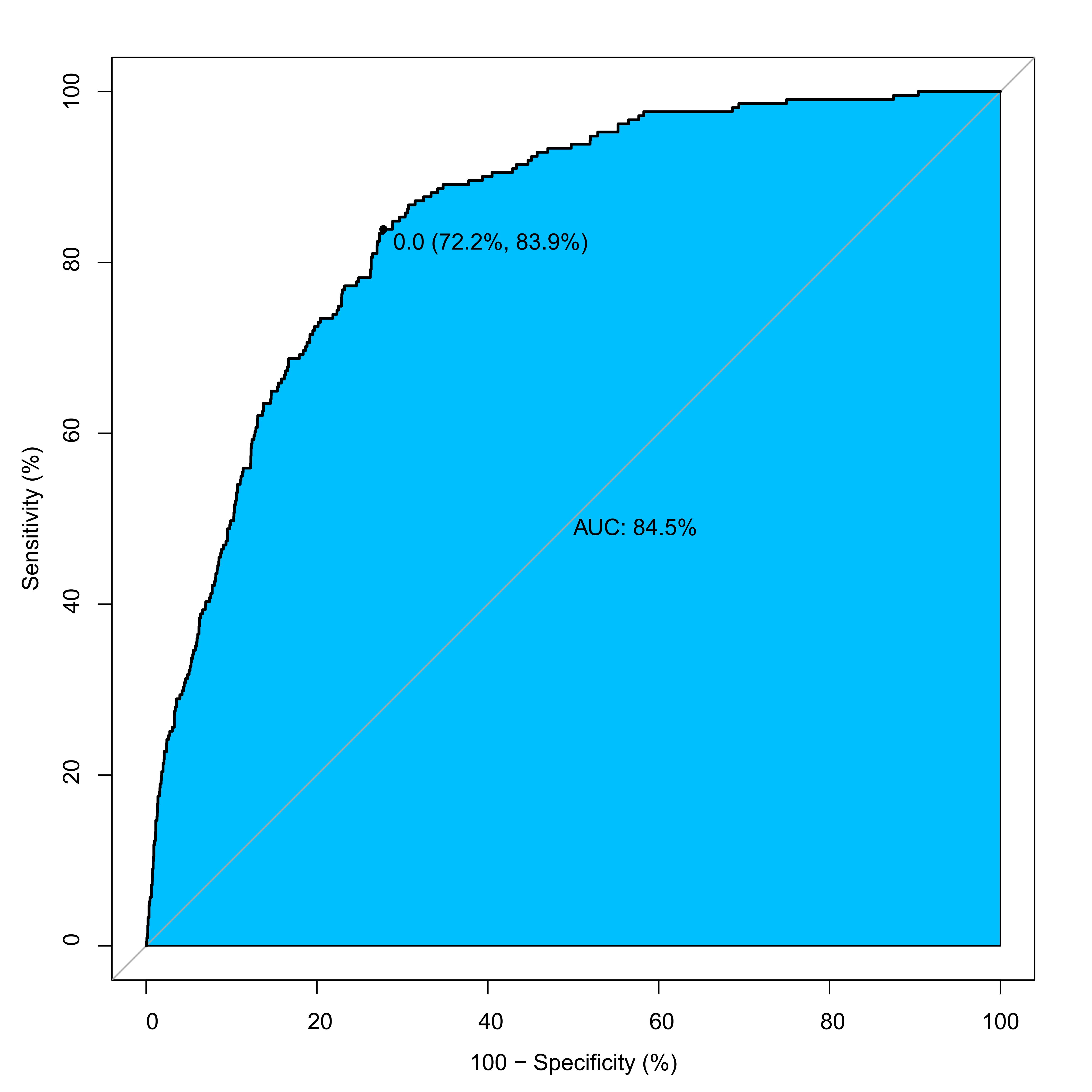

Supplement: Supplementary file 3 [file Image1.jpeg]

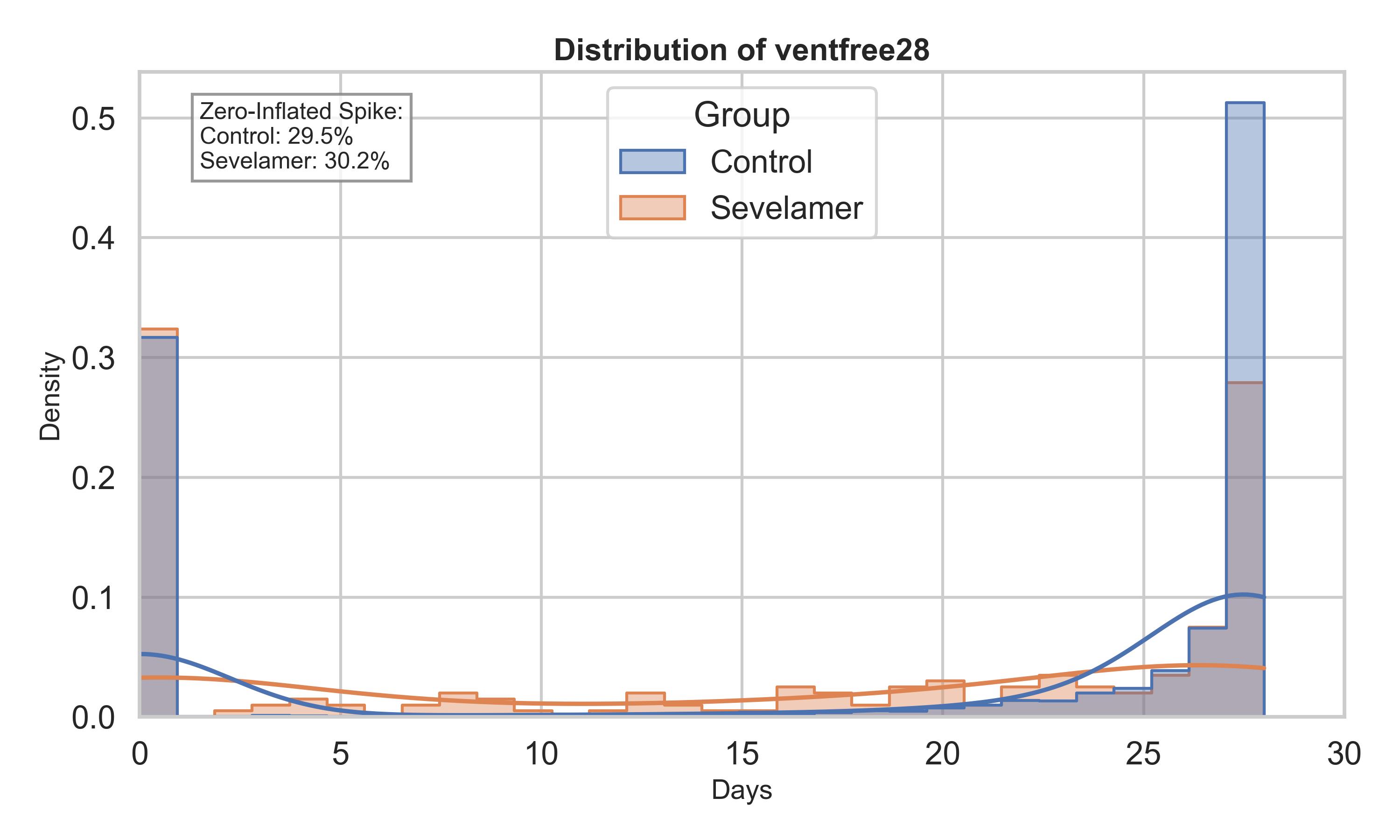

Supplement: Supplementary file 4 [file Image4.jpeg]

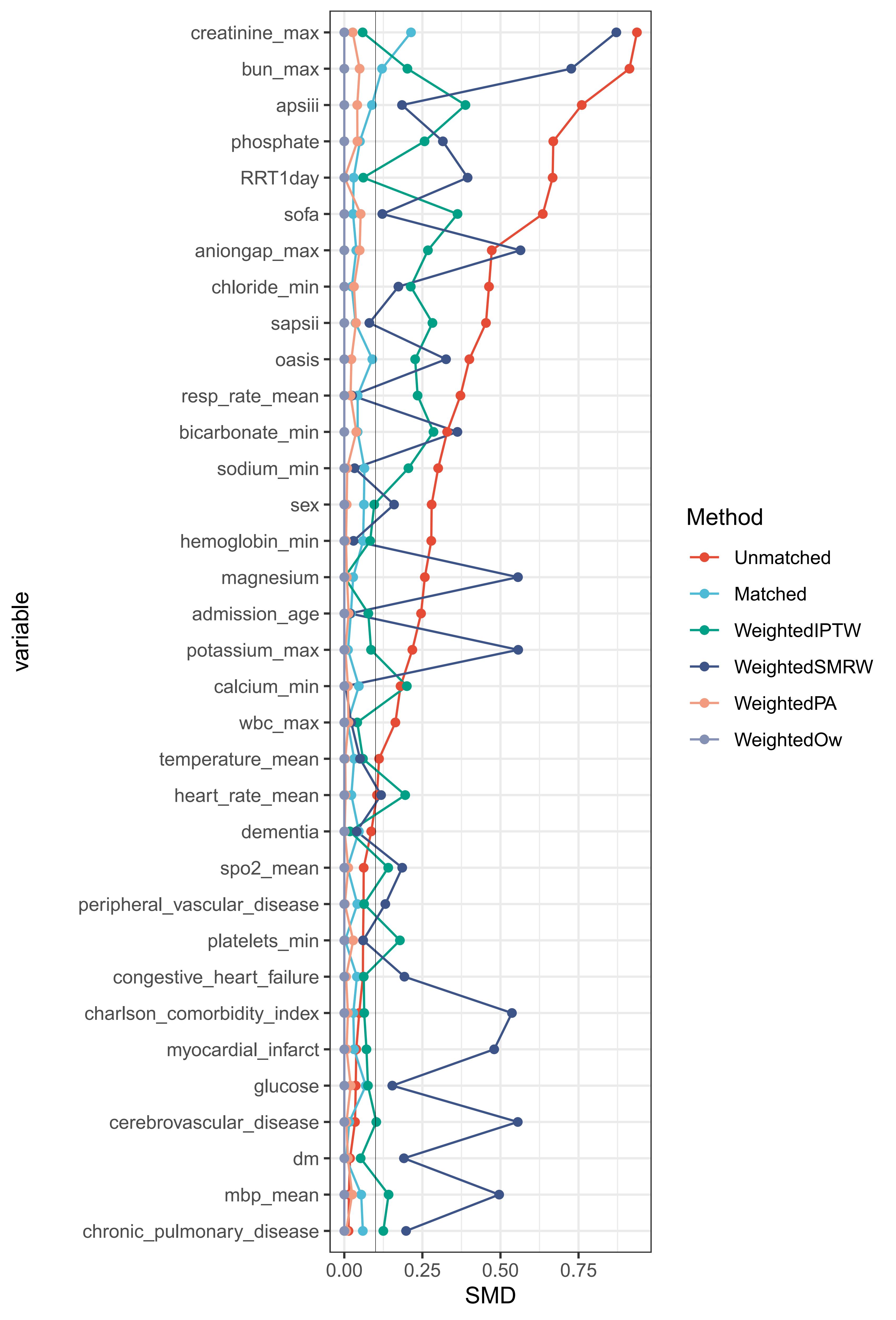

Supplement: Supplementary file 5 [file Image2.jpeg]

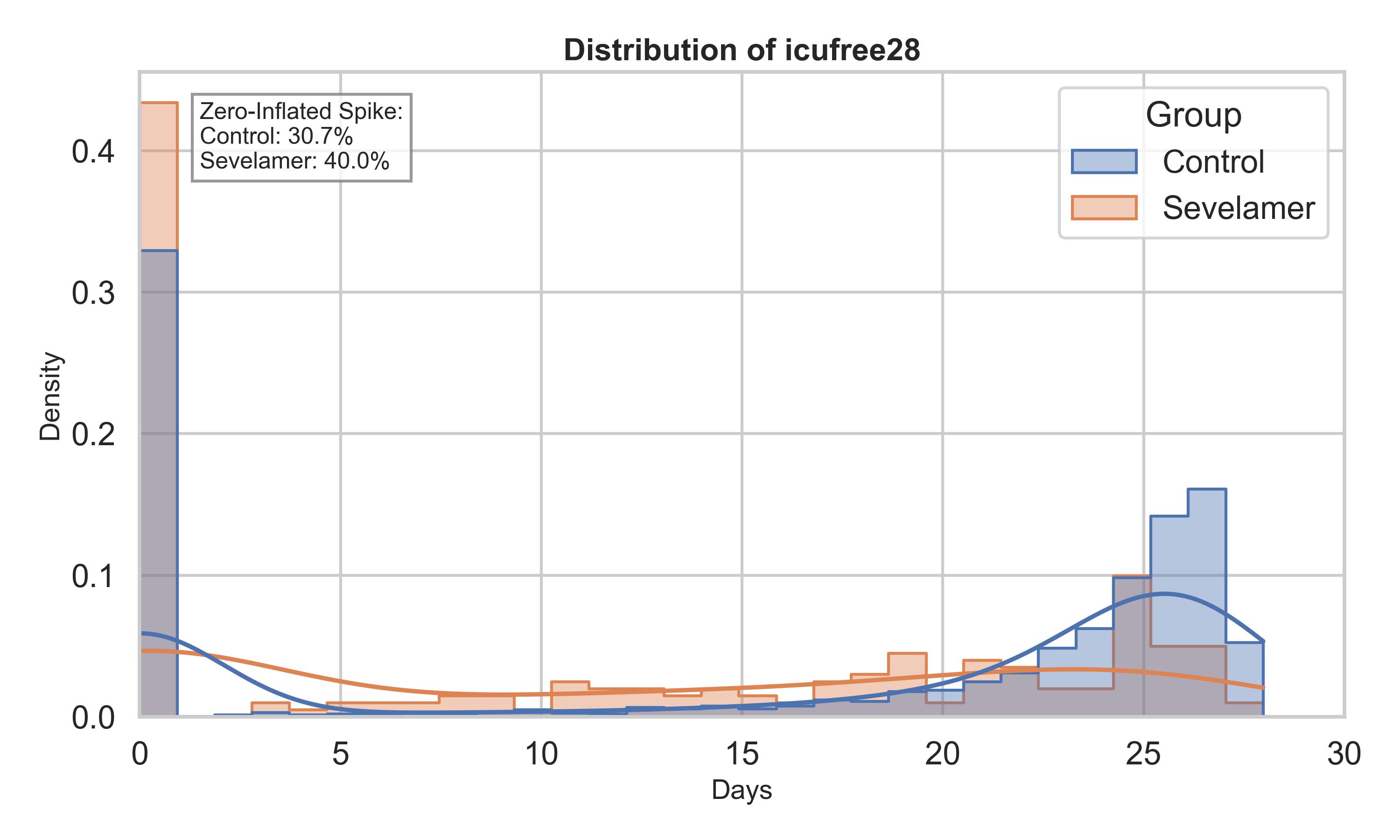

Supplement: Supplementary file 6 [file Image5.jpeg]
