# Supplementary material for: Association between sevelamer use and outcomes in acute kidney injury with hyperphosphataemia: evidence from the MIMIC-IV
Source: Front Pharmacol. 2026 Apr 29;17:1776446. doi: 10.3389/fphar.2026.1776446 (PMC13167594; doi:10.3389/fphar.2026.1776446)
Supplement: Supplementary file 2 [file Table1.docx]

Table 1 The variance inflation factors (VIFs) for multicollinearity assessment

| **Feature** | **VIF** |
| --- | --- |
| const | 5206.012710755831 |
| admission_age | 2.1772869700640967 |
| sex | 1.1238457424535513 |
| heart_rate_mean | 1.3960162749215 |
| mbp_mean | 1.2901262456375326 |
| resp_rate_mean | 1.5214919237277174 |
| temperature_mean | 1.2341610216371532 |
| spo2_mean | 1.2425865409036423 |
| hemoglobin_min | 1.353359508748781 |
| platelets_min | 1.348168085667987 |
| wbc_max | 1.0921872090573983 |
| aniongap_max | 5.294623190591243 |
| bicarbonate_min | 4.989553762874159 |
| bun_max | 2.363340828931505 |
| calcium_min | 1.3717830664049542 |
| chloride_min | 5.879651521448431 |
| creatinine_max | 2.2714599861414757 |
| sodium_min | 4.640605628422642 |
| potassium_max | 1.2545222200570196 |
| magnesium | 1.1511004641196185 |
| phosphate | 1.5855923482082517 |
| glucose_mean | 1.0103622484285788 |
| charlson_comorbidity_index | 2.07726467423557 |
| apsiii | 3.8184598135384844 |
| sapsii | 4.716388582168227 |
| oasis | 2.9978229148021733 |
| sofa | 3.6423824129827476 |
| dm | 1.1276768425595052 |
| myocardial_infarct | 1.1391383059195395 |
| congestive_heart_failure | 1.2146130304541227 |
| peripheral_vascular_disease | 1.0713471658336806 |
| cerebrovascular_disease | 1.0865215358640625 |
| chronic_pulmonary_disease | 1.13635201184237 |
| dementia | 1.0679867460625858 |

A strict threshold of VIF ≥ 10 was applied to exclude severely collinear variables.
