# Supplementary material for: Association between sevelamer use and outcomes in acute kidney injury with hyperphosphataemia: evidence from the MIMIC-IV
Source: Front Pharmacol. 2026 Apr 29;17:1776446. doi: 10.3389/fphar.2026.1776446 (PMC13167594; doi:10.3389/fphar.2026.1776446)
Supplement: Supplementary file 7 [file Table2.docx]

Table 2 The sensitivity analysis of early sevelamer exposure(≤72h) and outcomes using multivariable Cox regression

| **Variable** | **n.total** | **n.event_%** | **Followup.Time** | **crude.HR (95%CI)** | **crude.P value** | **adj.HR (95%CI)** | **adj.P value** |
| --- | --- | --- | --- | --- | --- | --- | --- |
| No Sevelamer | 4488 | 1190 (26.5) | 100751.4 | 1(Ref) |  | 1(Ref) |  |
| Sevelamer | 125 | 34 (27.2) | 2837.9 | 1 (0.71~1.41) | 0.993 | 0.69 (0.49~0.98) | 0.04 |

Two- sided P<0.05 denoted statistical significance.
